# Successful Endoscopic Management of Bouveret Syndrome by Transpyloric Stone Relocation and Intragastric Mechanical Lithotripsy

**DOI:** 10.3390/jcm15197612

**Published:** 2026-10-01

**Authors:** Mohamed El-Mahrouk, Emina Talakić, Robert Sucher, Sandro M. Hasenhütl

**Affiliations:** 1 Division of General, Visceral and Transplantation Surgery, Department of Surgery, Medical University of Graz, 8036 Graz, Austria; mohamed.el-mahrouk@medunigraz.at (M.E.-M.);; 2 Division of General Radiology, Department of Radiology, Medical University of Graz, 8036 Graz, Austria

**Keywords:** Bouveret syndrome, gallstone ileus, transpyloric stone relocation, gastric outlet obstruction, mechanical lithotripsy, therapeutic endoscopy

## Abstract

**Background:** Bouveret syndrome is a rare form of gallstone ileus in which a gallstone migrates through a bilioenteric fistula and impacts at the gastric outlet or proximal duodenum. It affects predominantly frail elderly women, in whom an endoscopy-first strategy is desirable; however, complete endoscopic clearance is achieved in fewer than half of cases and becomes markedly less likely as stone size increases, meaning that large stones often prompt surgery. **Case Presentation:** A 95-year-old woman presented with four days of vomiting and obstipation. Computed tomography demonstrated gastric outlet obstruction caused by a large gallstone impacted in the duodenal bulb with pneumobilia, consistent with Bouveret syndrome. Owing to her advanced age and operative risk, an entirely endoscopic approach was pursued. Initial extraction within the duodenal bulb using a retrieval net and foreign-body forceps failed because neither device could engage the stone. The closed forceps were therefore used as a lever to rotate and mobilize the stone until complete capture with the retrieval net became possible, after which the 37 mm stone was relocated through the pylorus into the stomach under controlled traction. As intact transoral extraction remained impossible, intragastric mechanical lithotripsy was performed and all fragments were retrieved transorally, achieving complete endoscopic clearance without surgery. The post-procedural course was uneventful; oral intake was resumed the following day, and the patient was discharged in good condition. **Conclusions:** Relocation of an impacted gallstone into the stomach before mechanical lithotripsy has been described previously. In this case, the stone could not initially be engaged; closed forceps used as a lever to rotate and mobilize it made capture with a retrieval net, and thus relocation, possible. This step allowed for complete endoscopic clearance without surgery in this single high-risk patient; its wider applicability remains to be established.

## 1. Introduction

Gallstone ileus is an uncommon cause of mechanical bowel obstruction, occurring in approximately 0.3–0.5% of patients with cholelithiasis [1]. Bouveret syndrome is its rarest and most proximal variant, in which a large gallstone migrates through a bilioenteric—most commonly cholecystoduodenal—fistula and becomes impacted in the duodenal bulb or gastric outlet, resulting in gastric outlet obstruction [2]. It accounts for only 1–3% of all cases of gallstone ileus [1]. Bouveret syndrome occurs predominantly in frail elderly women, with a reported mean age of 74 years and a female-to-male ratio of approximately 1.82:1 [2].

Because affected patients are typically elderly with multiple comorbidities, endoscopic retrieval—with or without lithotripsy—is generally recommended as the initial approach, with surgery (enterotomy or gastrotomy) reserved for endoscopic failure [2,3]. However, complete endoscopic clearance is achieved in only a minority of patients, with reported success rates of approximately 29–43% across systematic reviews [2,3]. This heterogeneity is driven substantially by stone size: in a post hoc validation cohort, clearance was achieved in 65.0% (13/20) of cases with stones ≤ 4 cm versus only 21.7% (5/23) with stones > 4 cm (odds ratio 6.7, 95% confidence interval 1.7–25.8), and the median diameter was 30 mm among endoscopically successful cases compared with 50 mm among those ultimately requiring surgery [4]. Obstructing stones—usually larger than 2–2.5 cm—generally require fragmentation before extraction, and multimodal endoscopic therapy (extraction combined with a lithotripsy technique) increases the likelihood of success [2,3,5]. Importantly, contemporary systematic-review data indicate that surgical mortality is substantially lower than the previously reported rates of up to 30%—approximately 6% in the largest series—which, together with the limited efficacy of endoscopy for large stones, has prompted calls for a more selective rather than routine endoscopic approach [3,6].

We report the successful entirely endoscopic management of Bouveret syndrome due to a large, impacted gallstone in a 95-year-old woman. In the present case, a 37 mm gallstone was mobilized from the duodenal bulb and intentionally relocated into the stomach to allow for controlled mechanical lithotripsy and complete transoral fragment retrieval. Transpyloric relocation of the intact stone followed by intragastric mechanical lithotripsy has previously been reported in three cases; in a fourth report, the stone was first fragmented in situ by laser lithotripsy and only its fragments reached the stomach [7,8,9,10]. This report details the step that preceded relocation in a stone that could not initially be engaged: the use of closed forceps as a lever to rotate and mobilize the stone until complete capture with a retrieval net became possible.

## 2. Case Presentation

### 2.1. Presentation and Diagnosis

A 95-year-old woman presented to the emergency department with a four-day history of vomiting and obstipation. Her medical history comprised arterial hypertension with a previous hypertensive crisis, coronary artery disease, mitral valve regurgitation, medication-induced coagulopathy, chronic bronchitis, degenerative spinal disease, and polyarthrosis; previous operations were a hysterectomy and a curettage. She was classified as American Society of Anesthesiologists (ASA) physical status III. She was hemodynamically stable on admission. Laboratory testing revealed a leukocytosis of 11.36 × 10^9^/L and a mildly elevated C-reactive protein of 9.2 mg/L; liver chemistry was otherwise unremarkable.

**Figure 1:**
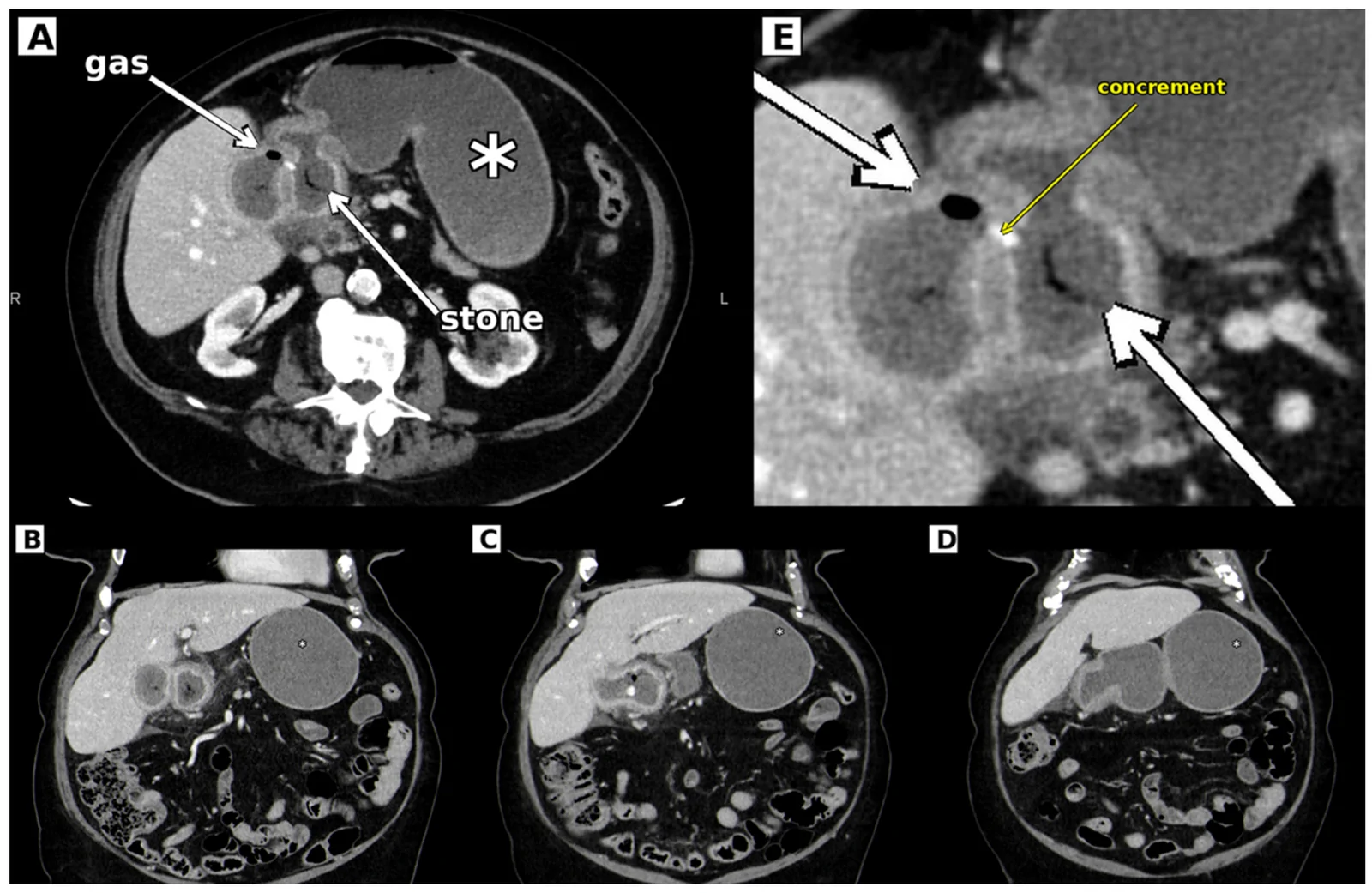
Contrast-enhanced axial computed tomography at presentation. (**A**) The stomach is markedly distended and fluid-filled (asterisk), indicating gastric outlet obstruction. Labeled arrows mark gas within the gallbladder and the ectopic gallstone impacted in the duodenal bulb. (**B**–**D**) Sequential axial images at progressively cranial levels demonstrate the markedly distended, fluid-filled stomach and duodenal bulb (asterisks). (**E**) Magnified view of panel A shows that only a small, discrete focus (arrow) within the impacted gallstone was clearly hyperdense; the remainder of the calculus was isodense to soft tissue.

Bedside ultrasonography demonstrated to-and-fro (pendulum) gastric peristalsis extending to the duodenum together with cholecystolithiasis, prompting computed tomography (CT). Contrast-enhanced CT demonstrated a markedly distended, fluid-filled stomach indicating gastric outlet obstruction, caused by a large ectopic gallstone impacted in the duodenal bulb. Pneumobilia, including gas within the gallbladder and biliary tree, was present, and the cholecystoduodenal fistulous tract was directly visualized. On CT, the calculus was largely isoattenuating to soft tissue, with only a small discrete hyperdense focus, so that it was not densely calcified radiologically despite its exceptional hardness at endoscopy. Because of this low attenuation, the maximal diameter of the calculus could not be measured reliably on CT; it was determined ex vivo from the retrieved fragments. These findings were consistent with Bouveret syndrome (Figure 1). A nasogastric tube was inserted for decompression before endoscopic therapy.

### 2.2. Endoscopic Procedure

**Figure 2:**
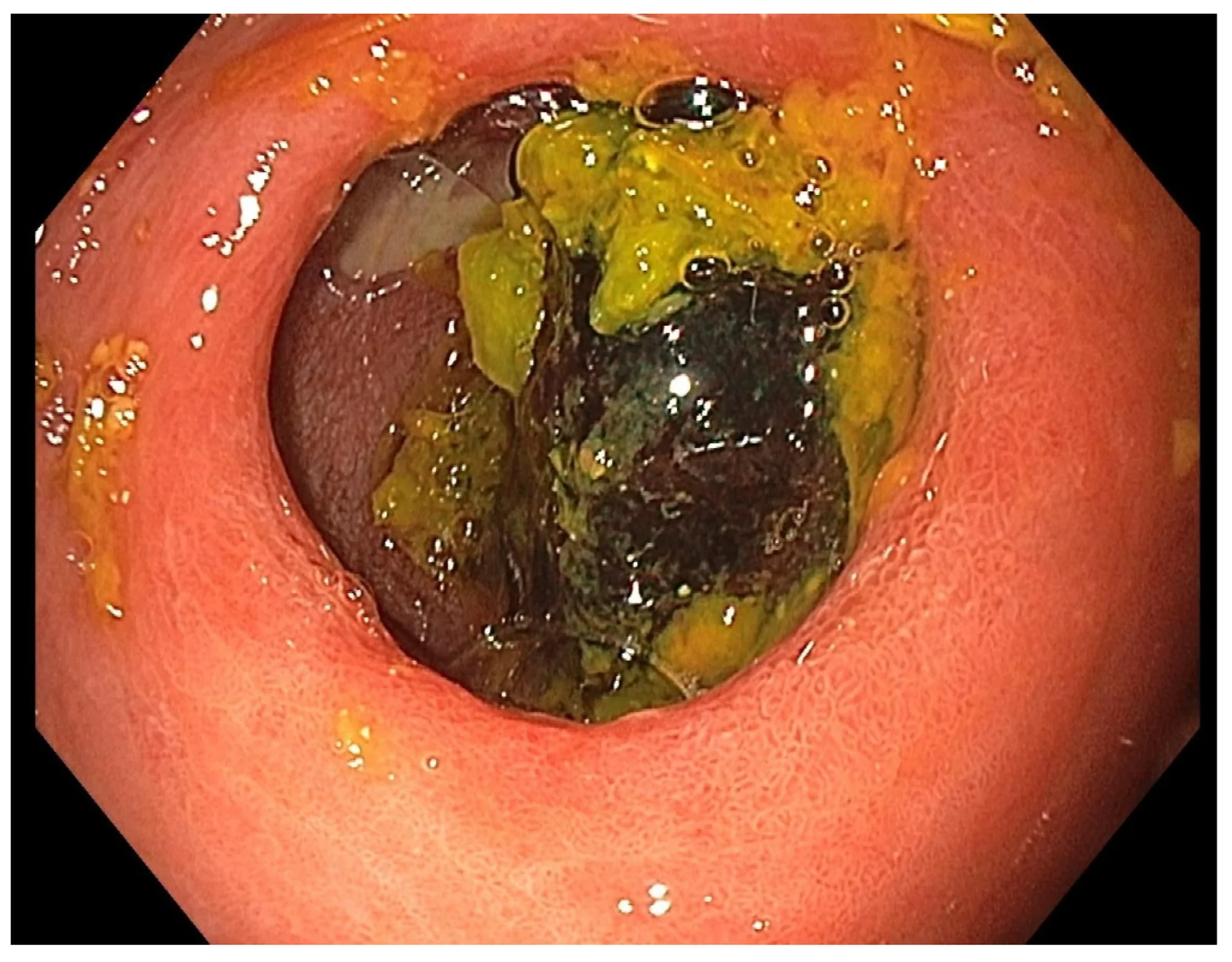
Endoscopic view from the gastric antrum demonstrating a large gallstone impacted in the duodenal bulb, causing gastric outlet obstruction.

Given the patient’s advanced age and substantial operative risk, an entirely endoscopic approach was planned under general anesthesia. Upper gastrointestinal endoscopy revealed a large gallstone completely impacted within the duodenal bulb, causing gastric outlet obstruction (Figure 2). The diagnostic gastroscope could not be advanced beyond the impacted stone.

**Figure 3:**
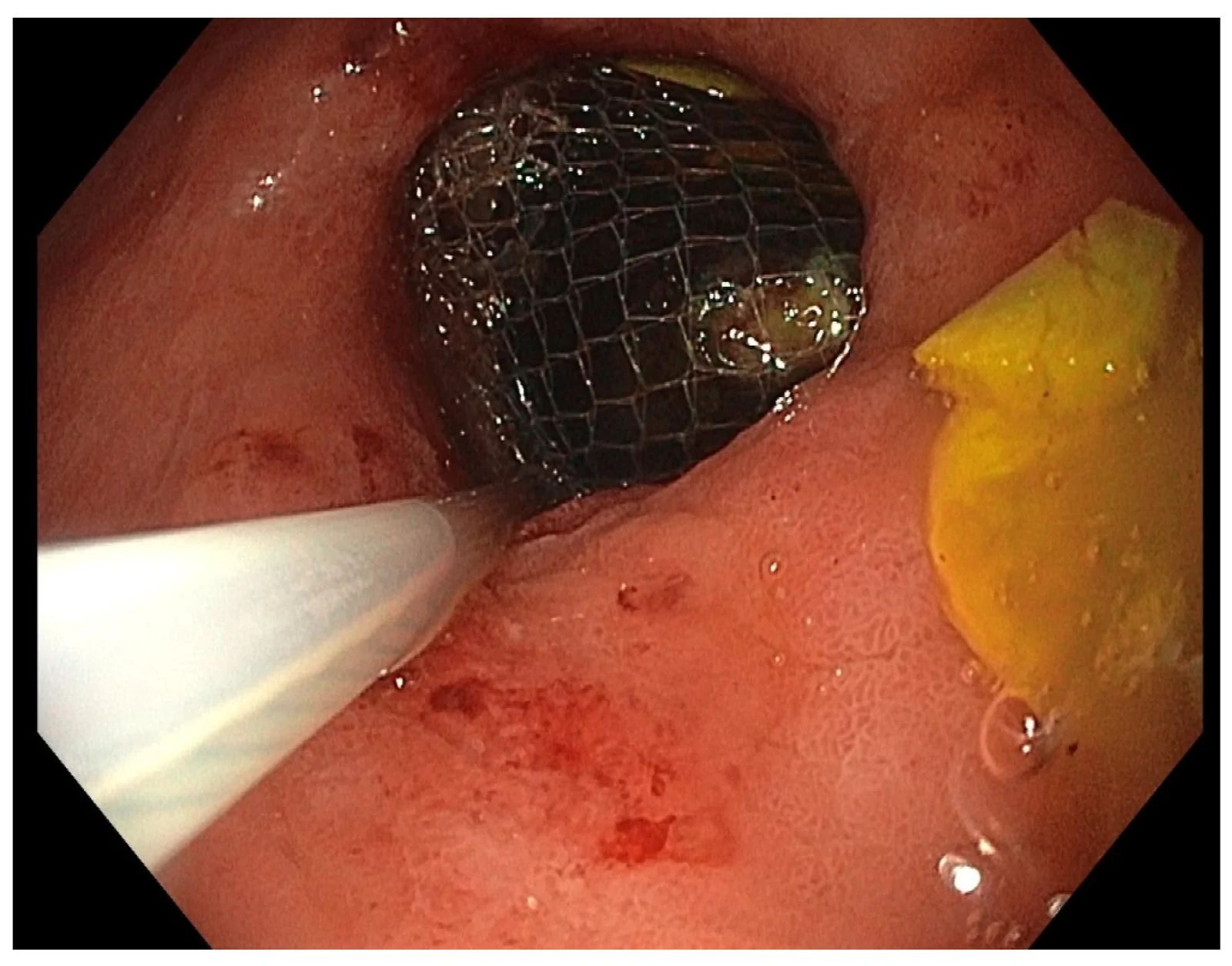
Mobilized gallstone completely captured with a Roth Net™ retrieval device in the duodenal bulb before transpyloric relocation. The retrieval net was aligned coaxially with the pyloric channel to establish a straight, controlled traction vector towards the greater curvature of the stomach.

**Figure 4:**
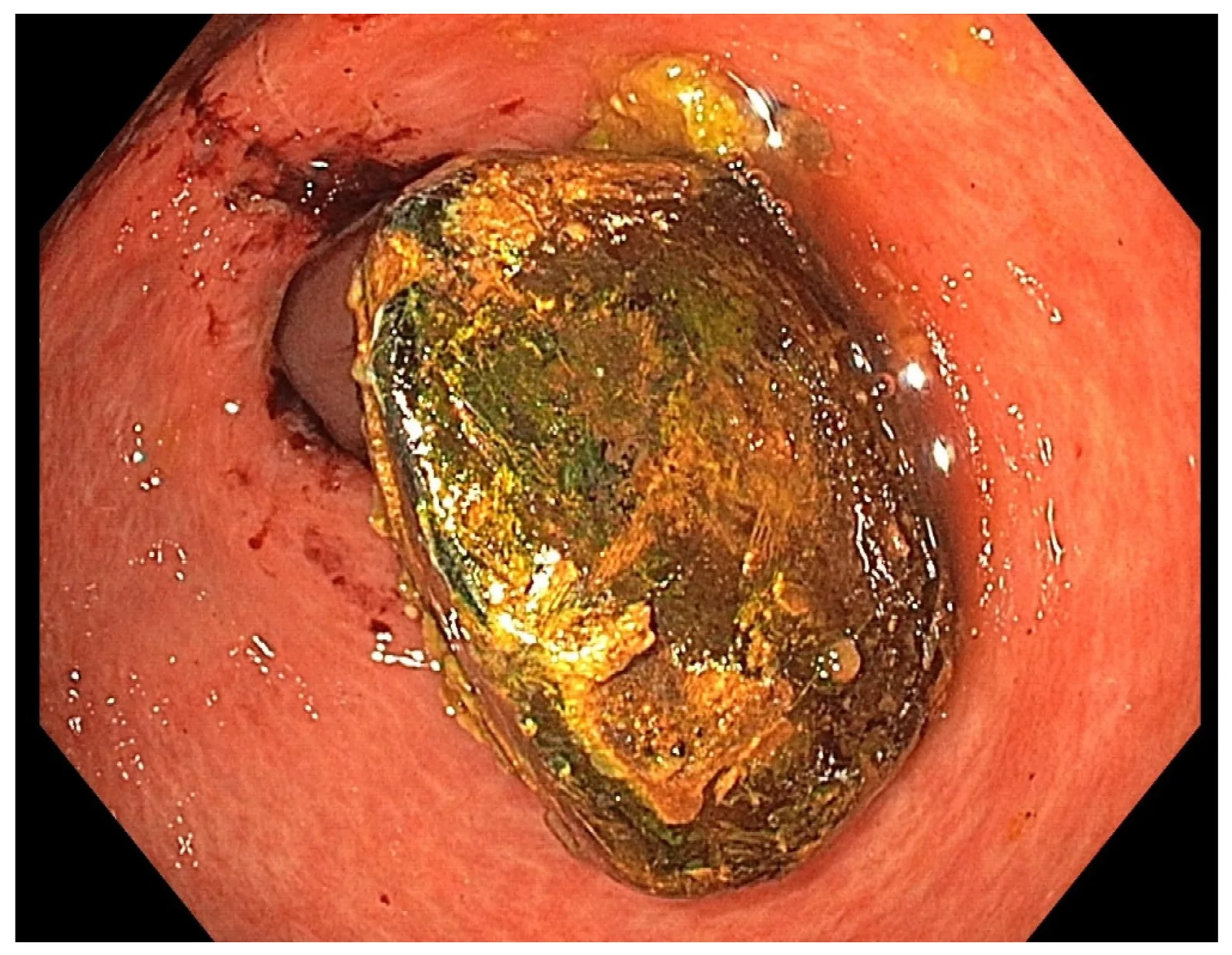
The gallstone after successful transpyloric relocation into the gastric antrum.

Initial attempts at endoscopic extraction using a retrieval net were unsuccessful because the stone could not be completely engaged. Owing to its exceptional hardness and smooth surface, foreign-body forceps failed to obtain sufficient purchase. The closed forceps were therefore introduced transpylorically and used as a lever rather than as a grasping device. By applying gentle pressure to the exposed surface of the stone, the calculus was carefully rotated and mobilized within the duodenal bulb until it could be completely captured with the retrieval net (Roth Net™ polyp retrieval device, nominal net size 3 × 6 cm; STERIS, Mentor, OH, USA; Figure 3). While maintaining net capture, the endoscope and retrieval net were positioned to create a straight traction vector aligned with the longitudinal axis of the pyloric channel. This vector was directed towards the greater curvature of the stomach, thereby avoiding excessive focal traction on the pyloric rim. Controlled traction was then increased gradually until the stone passed through the pylorus and was relocated into the gastric antrum. Once relocated into the stomach, the gallstone was visible in its entirety and proved irregular and asymmetric, in contrast to the smooth aspect that had presented within the bulb (Figure 4).

Because intact transoral extraction remained impossible due to the stone’s size, several additional retrieval strategies were attempted. Neither the use of a foreign-body retrieval cap nor the additional exchange to a therapeutic gastroscope enabled retrograde passage of the stone across the cardia. Likewise, attempted fragmentation using a polypectomy snare proved unsuccessful because of the stone’s extreme hardness, and a Dormia basket (3.0 cm Trapezoid™ RX retrieval basket; Boston Scientific, Marlborough, MA, USA) was too small to fully capture the gallstone.

**Figure 5:**
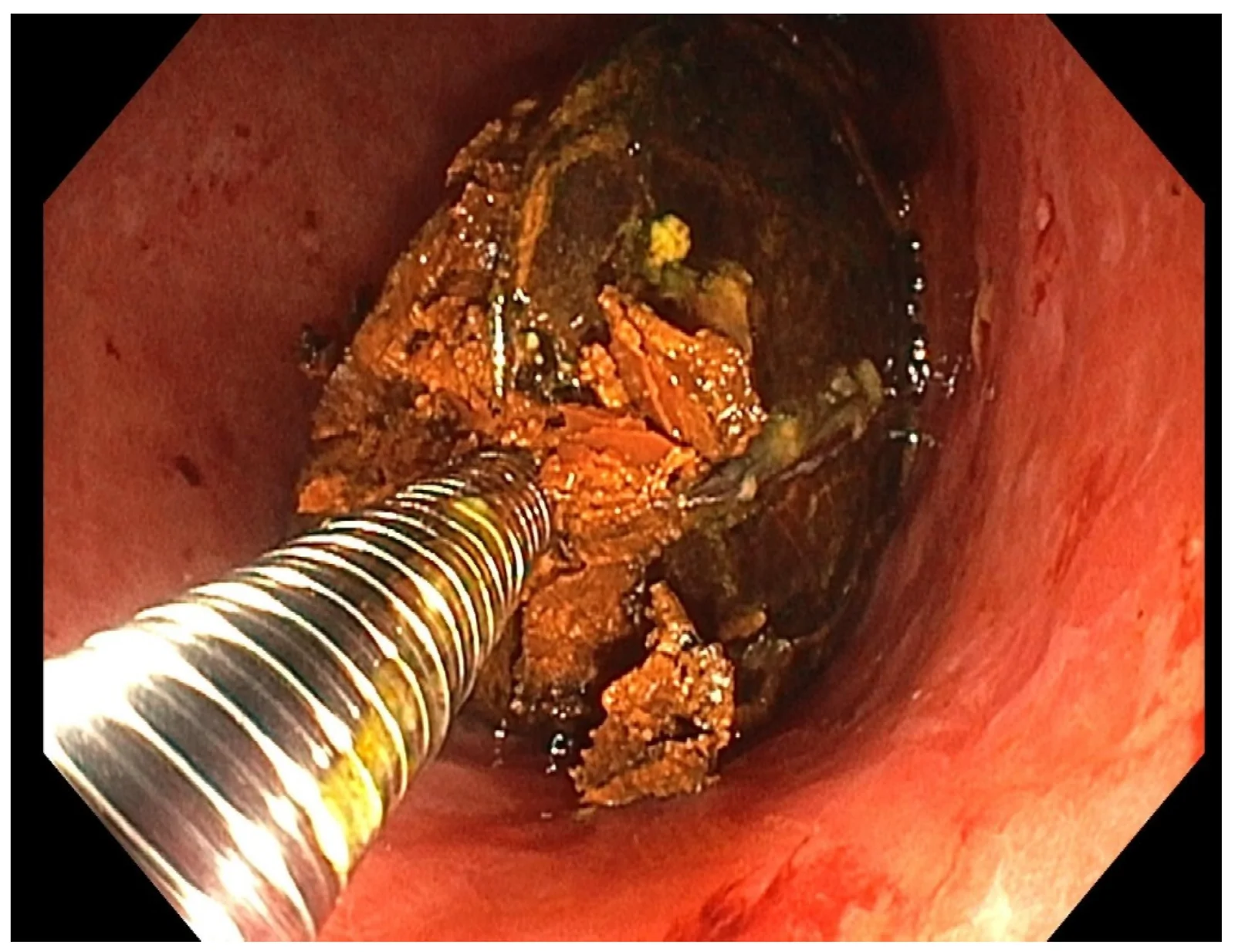
The relocated gallstone engaged within the mechanical lithotripter at the beginning of mechanical lithotripsy.

**Figure 6:**
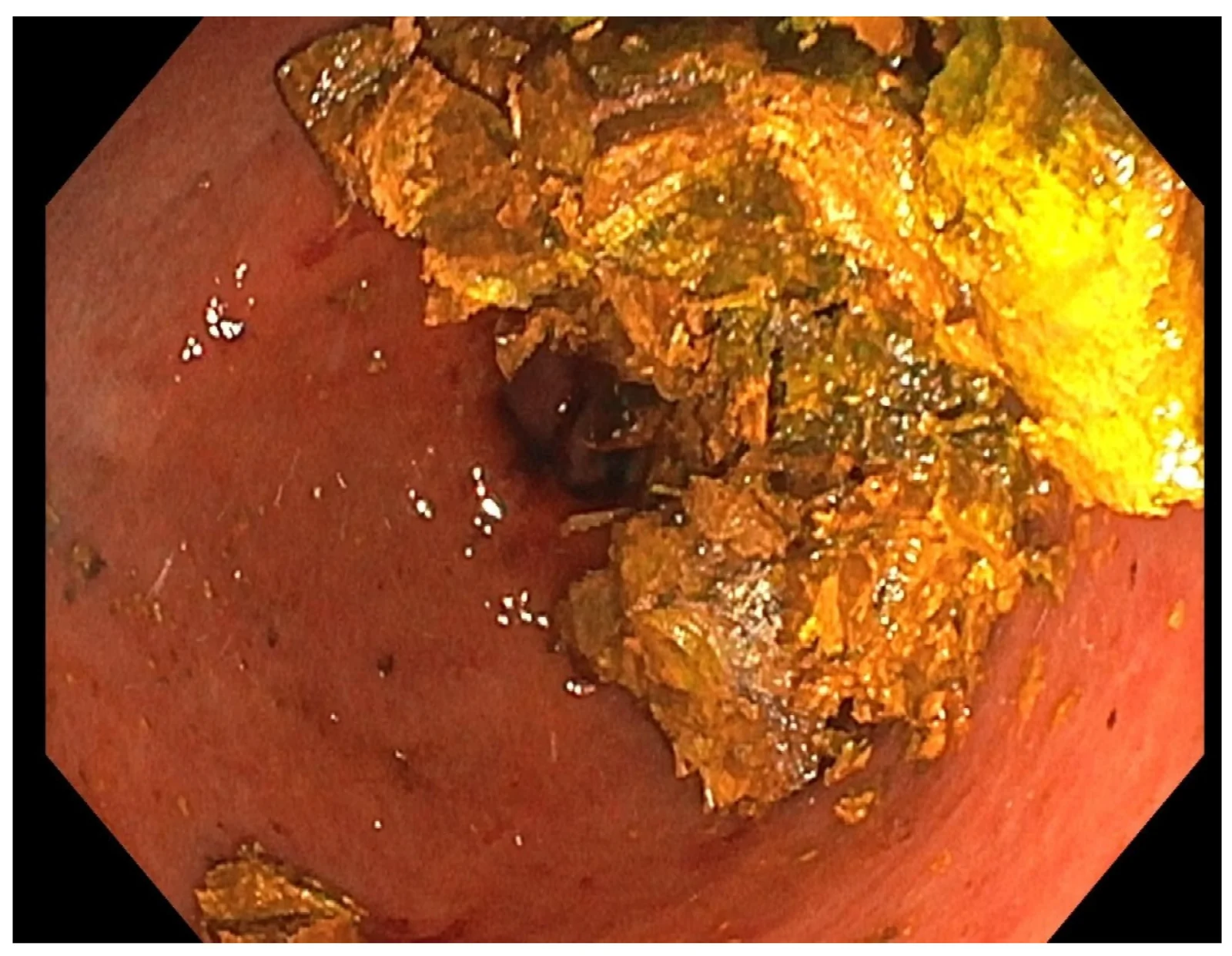
Gallstone fragments in the gastric antrum following successful mechanical lithotripsy.

**Figure 7:**
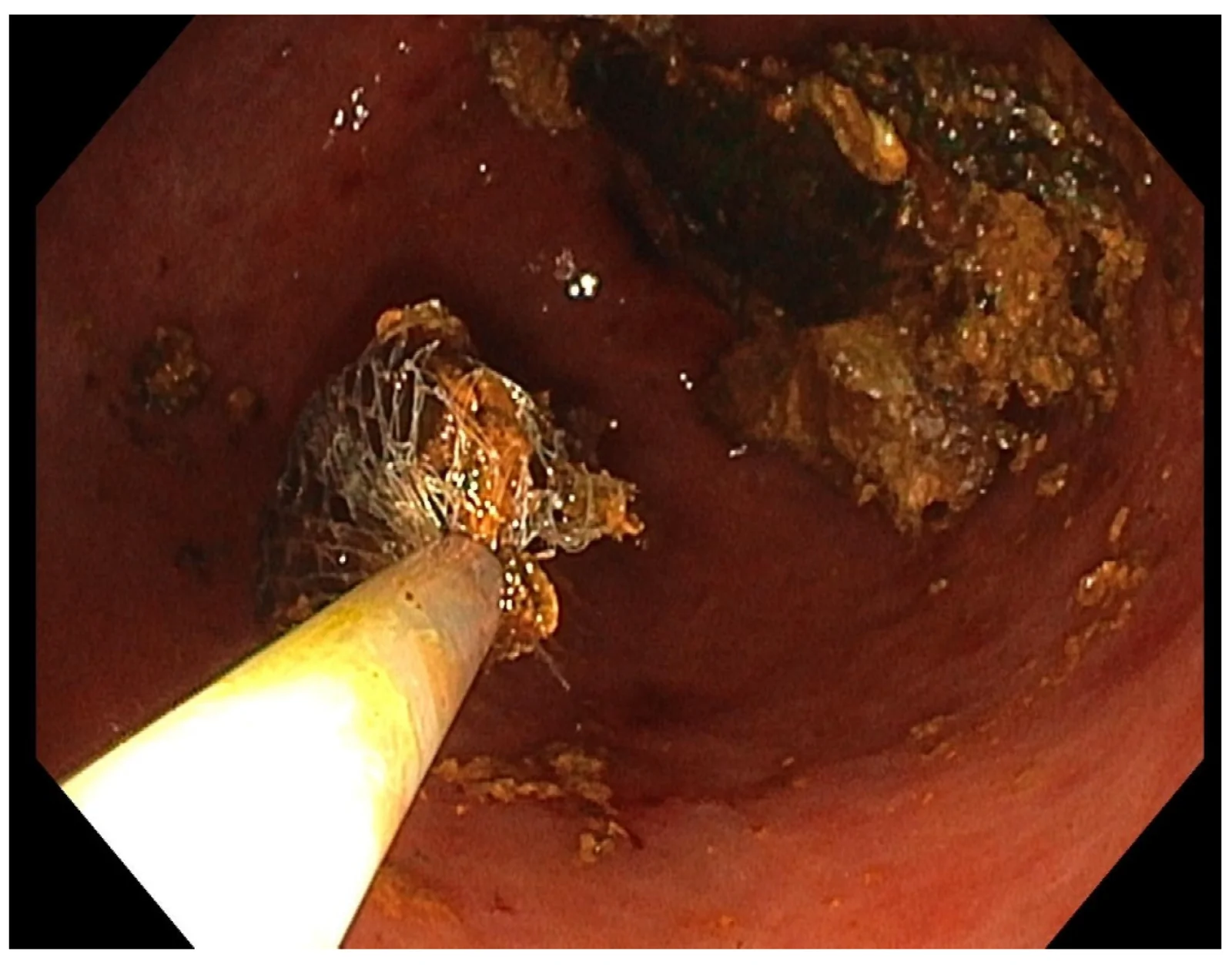
Retrieval of a lithotripsy fragment with a retrieval net before transoral extraction.

Rather than attempting mechanical lithotripsy within the confined duodenal bulb, definitive fragmentation was intentionally performed after relocation into the stomach, where the substantially larger working space permitted controlled positioning of the lithotripter around the gallstone. Ultimately, a 30 mm LithoCrush V mechanical lithotripter (Olympus, Tokyo, Japan) could be advanced over the stone, and gradual tightening of the device resulted in complete fragmentation over approximately 10 min (Figure 5). All stone fragments were subsequently removed transorally using the Roth Net™ retrieval device (Figure 6 and Figure 7). To minimize the risk of mucosal injury, fragments were first reduced to a size suitable for transoral retrieval and fully enclosed within the retrieval net. Withdrawal was performed deliberately slowly under continuous endoscopic visualization, maintaining a constant view of the retrieved fragment and its interaction with the surrounding mucosa throughout passage of the upper gastrointestinal tract. Generous insufflation was maintained during the withdrawal to maximize luminal distension and thereby minimize contact pressure. Fragment retrieval was performed without resistance. After completion of extraction, the cardia, esophagus, and upper esophageal sphincter were carefully inspected endoscopically, with no evidence of procedure-related mucosal injury.

**Figure 8:**
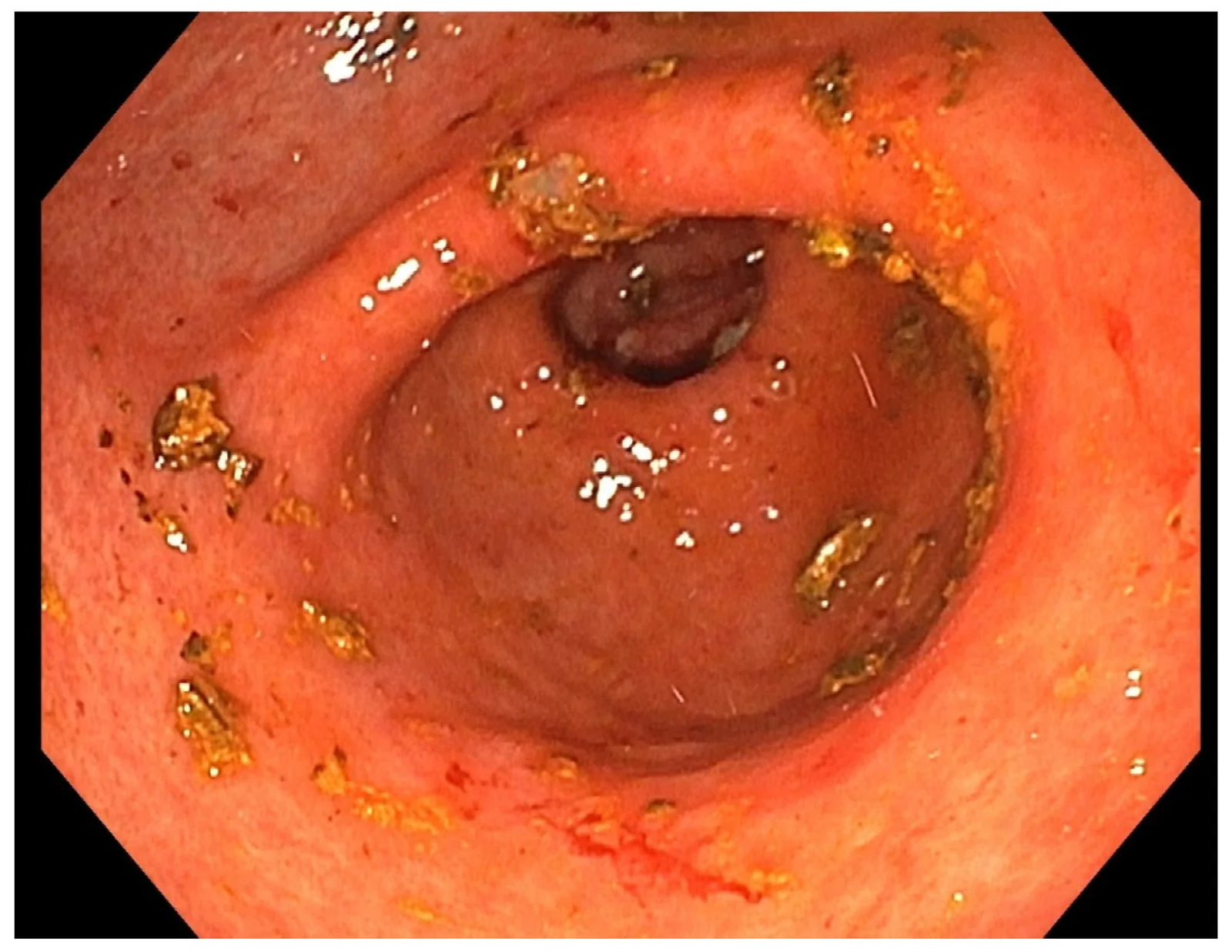
Gastric antrum after complete extraction of all lithotripsy fragments.

**Figure 9:**
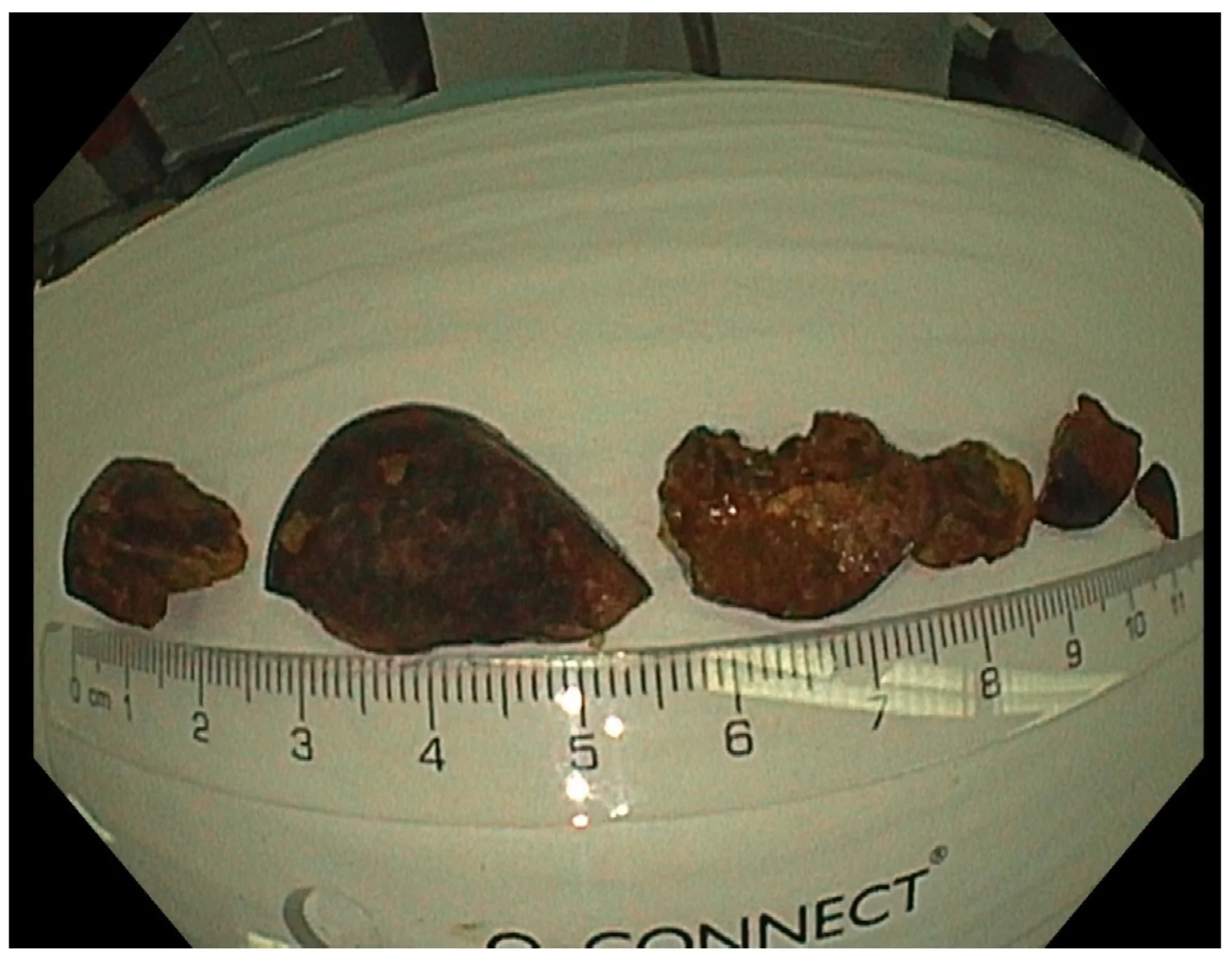
Retrieved gallstone fragments shown with a ruler for size reference.

Final endoscopic inspection demonstrated complete clearance of the gastric lumen and duodenal bulb, with no residual stone fragments identified endoscopically (Figure 8); the retrieved fragments are shown in Figure 9. The maximal stone diameter, determined ex vivo from the retrieved fragments, was 37 mm. Pressure-related fibrin deposition was present at the previous impaction site. Although a cholecystoduodenal fistulous tract had been directly visualized on CT, no discrete fistulous orifice could be identified endoscopically. The endoscope was advanced into the descending duodenum without difficulty. The total endoscopic procedure time was 89 min. As luminal patency had been fully restored, replacement of the nasogastric tube was not required, and the procedure was completely uneventful.

### 2.3. Post-Procedural Course

The post-procedural course was uneventful. On the first post-procedural day, the leukocyte count had normalized, while the C-reactive protein showed only a minimal transient increase to 13.7 mg/L. Oral intake was resumed without difficulty, and no procedure-related complications occurred. The patient remained clinically stable and was discharged in good general condition five days after presentation (three days after the endoscopic intervention).

Given the patient’s advanced age, substantial comorbidity burden, complete resolution of the gastric outlet obstruction, absence of biliary symptoms, and uneventful post-procedural recovery, no scheduled endoscopic or imaging surveillance was pursued. Further invasive assessment of the cholecystoduodenal fistula was considered unlikely to alter management in this 95-year-old patient. Follow-up was therefore symptom-driven rather than protocolized, and no structured follow-up data are available.

## 3. Discussion

Bouveret syndrome predominantly affects elderly patients with substantial comorbidity, in whom surgical morbidity has traditionally made endoscopy the preferred first-line approach, although complete endoscopic clearance is achieved in only a minority of cases [2,3]. In the biliary literature, stone impaction and a restricted, acutely angulated working segment—rather than stone size alone—have been identified as determinants of failed mechanical lithotripsy and incomplete duct clearance [11,12].

In our patient, relocation of the calculus from the duodenal bulb into the substantially more spacious gastric lumen allowed the lithotripter to be positioned around the stone and all fragments to be retrieved under direct vision. This may additionally reduce the risk of distal migration of fragments, which can result in secondary small-bowel gallstone ileus [3]. This single case cannot establish restricted working space as a general determinant of endoscopic failure.

Relocation of an impacted gallstone into the stomach followed by mechanical lithotripsy has been reported previously in three cases. Doycheva et al. reported successful transfer of a 3.3 cm gallstone from the second portion of the duodenum into the stomach using various devices, including retrieval net, snare, stone-extraction basket, and lithotripsy basket, followed by mechanical lithotripsy [7]. Dixon et al. secured a 2 × 3 cm gallstone impacted in the duodenal angle with a 3 × 6 cm retrieval device, followed by intragastric mechanical lithotripsy and transoral fragment removal [8]. Hsu et al. grasped a 3 cm stone in the duodenal bulb with a 3.5 cm mechanical lithotripsy basket, achieved passage into the stomach with two minutes of constant traction, and then performed intragastric mechanical lithotripsy with retrieval of the fragments by Roth net, proposing traction-assisted relocation as a technique in its own right [9]. A related report differs in that the stone was not relocated intact: Rogart et al. reported that, for a 3 × 2.5 cm stone impacted in the duodenal bulb, there was “no room to maneuver a mechanical lithotripter around the stone”, so that holmium:yttrium-aluminum-garnet laser lithotripsy had to be applied in situ and mechanical lithotripsy became feasible only once fragments had reached the stomach [10].

At 37 mm, the stone in our patient was larger than those in these reports and lay between the median diameter of endoscopically cleared stones (30 mm) and that of stones ultimately requiring surgery (50 mm) in the largest validation cohort [4], and therefore in a size range in which the outcome of endoscopic therapy is not readily predictable. The main difference from the previous reports lies in the step preceding relocation. In the three cases in which an intact stone was drawn through the pylorus, it had first been directly engaged with a basket or retrieval device [7,8,9]. In our case, primary capture was not possible as the retrieval net could not be closed around the stone and foreign-body forceps obtained no purchase on its smooth surface. Capture, the prerequisite for any traction maneuver, therefore had to be created first. Closed forceps were used as a lever to gently rotate and mobilize the impacted stone, permitting complete capture with a retrieval net.

After relocation, definitive fragmentation was achieved using a mechanical lithotripter. This carries a practical advantage: mechanical lithotripsy is widely available and does not require laser or electrohydraulic lithotripsy equipment or specialized infrastructure, which may be limited to tertiary centers [9,13,14]. Mechanical lithotripsy achieves stone clearance in 76–91% of biliary cases [13,14]. In a series of 134 patients with difficult biliary stones, stone impaction was the strongest predictor of lithotripsy failure (odds ratio 17.83), ahead of stone size above 30 mm and an unfavorable stone-to-duct diameter ratio [14]. In the present case, the stone resisted forceps manipulation while impacted in the duodenal bulb and could not be fragmented by snare or basket even after transpyloric relocation. In the stomach, however, mechanical lithotripsy was readily achieved once the lithotripter could be positioned around the stone under direct endoscopic visualization.

Several limitations must be acknowledged. This is a single case report, and no general recommendation can be derived from it. The stone diameter was determined ex vivo from the retrieved fragments, as the largely isoattenuating calculus could not be measured reliably on CT. The feasibility of transpyloric relocation depends on stone size, its mobility, and the individual pyloroduodenal anatomy. A firmly impacted or immobile stone may not be suitable for this maneuver, and excessive force may cause pyloric or bulbar injury. The approach also requires advanced interventional endoscopic expertise and should be reserved for experienced endoscopists and centers able to manage potential complications and provide surgical rescue if required. Additional reports and larger series are needed to better define appropriate patient selection.

Importantly, endoscopic treatment achieved complete stone clearance and resolution of the gastric outlet obstruction but did not address the underlying bilioenteric fistula. In the present case, the cholecystoduodenal fistulous tract was directly visualized on CT; however, no discrete endoluminal orifice was identified after stone removal. As endoscopy was performed with forward-viewing gastroscopes, visualization of the immediate postpyloric region and of a small or tangentially oriented fistulous orifice may have been limited. In addition, the opening may have collapsed after stone passage or been obscured by local edema and pressure-related fibrin deposition at the previous impaction site. Given the patient’s age of 95 years, substantial comorbidity burden, absence of biliary symptoms, and uneventful recovery, no further invasive assessment or treatment of the fistula was pursued. In this individual patient, the potential additional procedural burden of fistula-directed intervention was considered to outweigh the uncertain clinical benefit in the absence of persistent or recurrent symptoms. Management was therefore conservative, which is consistent with common practice in frail elderly patients, in whom the fistula is often observed and may close spontaneously [6]. Follow-up was symptom-driven rather than protocolized. Consequently, spontaneous fistula closure cannot be documented, and the absence of structured long-term follow-up represents a limitation of this report.

## 4. Conclusions

Bouveret syndrome caused by a large, impacted gallstone was successfully managed entirely endoscopically by intentional transpyloric stone relocation, intragastric mechanical lithotripsy, and complete transoral fragment retrieval. Because relocation followed by intragastric lithotripsy has been described previously, the contribution of this case lies in the step that made relocation possible: as the stone could not be engaged by retrieval net or foreign-body forceps, the closed forceps were used as a lever to rotate and mobilize it until complete capture with the retrieval net was achieved. As this is a single case, the step cannot be generalized, but it may be considered when an impacted gallstone cannot be captured by conventional means.

## 5. Learning Points

- When an impacted gallstone cannot be engaged by a retrieval net or foreign-body forceps, closed forceps used as a lever to rotate and mobilize the stone may allow for complete capture with a retrieval net, the prerequisite for transpyloric relocation into the stomach and subsequent intragastric mechanical lithotripsy.
- Mechanical lithotripsy alone sufficed to fragment a 37 mm stone once it had been moved into the stomach, without adjunctive laser or electrohydraulic energy—relevant wherever those platforms are unavailable.
- Endoscopic treatment resolves the obstruction but does not address the underlying bilioenteric fistula, and fragments must be retrieved rather than left to pass, to avoid distal gallstone ileus; multidisciplinary discussion and appropriate clinical follow-up remain essential.

## Data Availability

The data supporting the findings of this case report are available from the corresponding author upon reasonable request.
